# Supplementary material for: Adiponectin Enhances Fatty Acid Signaling in Human Taste Cells by Increasing Surface Expression of CD36
Source: Int J Mol Sci. 2023 Mar 18;24(6):5801. doi: 10.3390/ijms24065801 (PMC10059208; doi:10.3390/ijms24065801)
Supplement: Supplementary file 1 [file ijms-24-05801-s001.zip › ijms-2274755-supplementary.pdf]

International Journal of *Molecular Sciences*

MDPI

Supplementary Material to

Adiponectin Enhances Fatty Acid Signaling in Human Taste Cells by Increasing Surface Expression of CD36

Fangjun Lin [1,†], Yan Liu [2,†], Trina Rudeski-Rohr [1], Naima Dahir [1], Ashley Calder [1] and Timothy A. Gilbertson [2,*]

[1] Burnett School of Biomedical Sciences, College of Medicine, University of Central Florida, Orlando, FL 32827, USA; fangjun.lin@ucf.edu (F.L.); trudeski@knights.ucf.edu (T.R.-R.); ndahir@umich.edu (N.D.); ascalder@med.umich.edu (A.C.)
[2] Department of Internal Medicine, College of Medicine, University of Central Florida, Orlando, FL 32827, USA; liu.yan.7605@gmail.com
* Correspondence: timothy.gilbertson@ucf.edu; Tel.: +1-407-266-7245
† These authors contributed equally to this work.

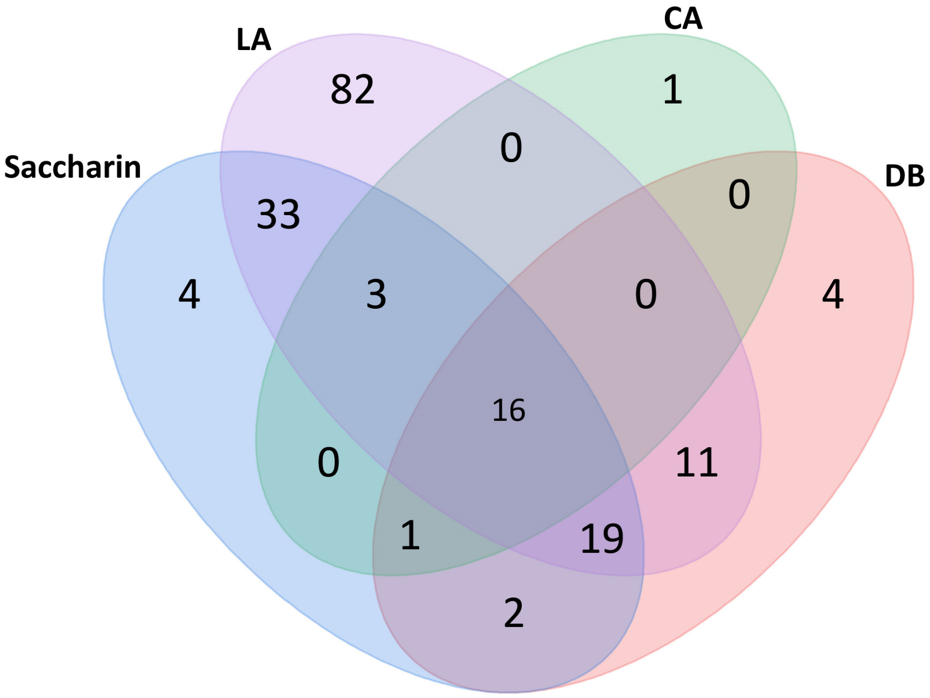

Figure S1. Venn diagram showing the number of HuFF cells responses to different tastants: saccharin (20 mM), denatonium benzoate (DB, 5 mM), linoleic acid (LA, 30 μM), and capric acid (CA, 100 μM) when applying these tastants. on the same cells. Because of the low number of responses to monosodium glutamate (MSG, 4 of 280 cells), this tastant was not included in the diagram.

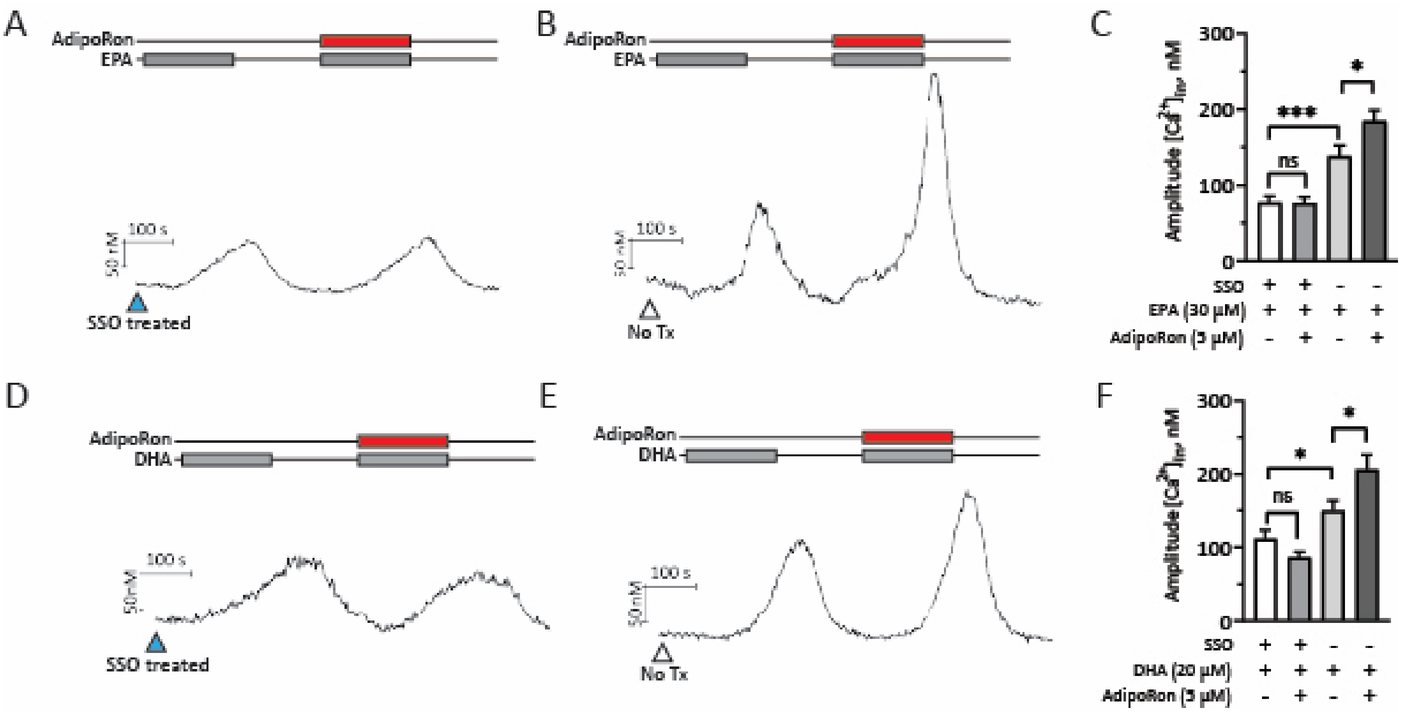

Figure S2. AdipoRon enhances cellular responses to EPA and DHA in HUFF cells. Representative calcium trace showing responses to 30 µM EPA with or without 5 µM AdipoRon in SSO pre-treated (***A***) or untreated (***B***) HuFF cells. ***C***, Mean effect of SSO, AdipoRon, and SSO + AdipoRon on calcium responses to 30 µM EPA. Representative calcium trace showing responses to 20 µM DHA with or without 5 µM AdipoRon in SSO pre-treated (***D***) or untreated (***E***) HuFF cells. ***F***, Effect of SSO, AdipoRon, and SSO + AdipoRon on calcium responses to 20 µM DHA. Similar to the case for LA, blocking CD36 eliminated the ability of AdipoRon to enhance calcium responses to EPA and DHA. Data are presented as mean ± SEM. Unpaired student's t-tests were used to determine statistical significance (ns $p > 0.05$, * $p < 0.05$, *** $p < 0.001$).

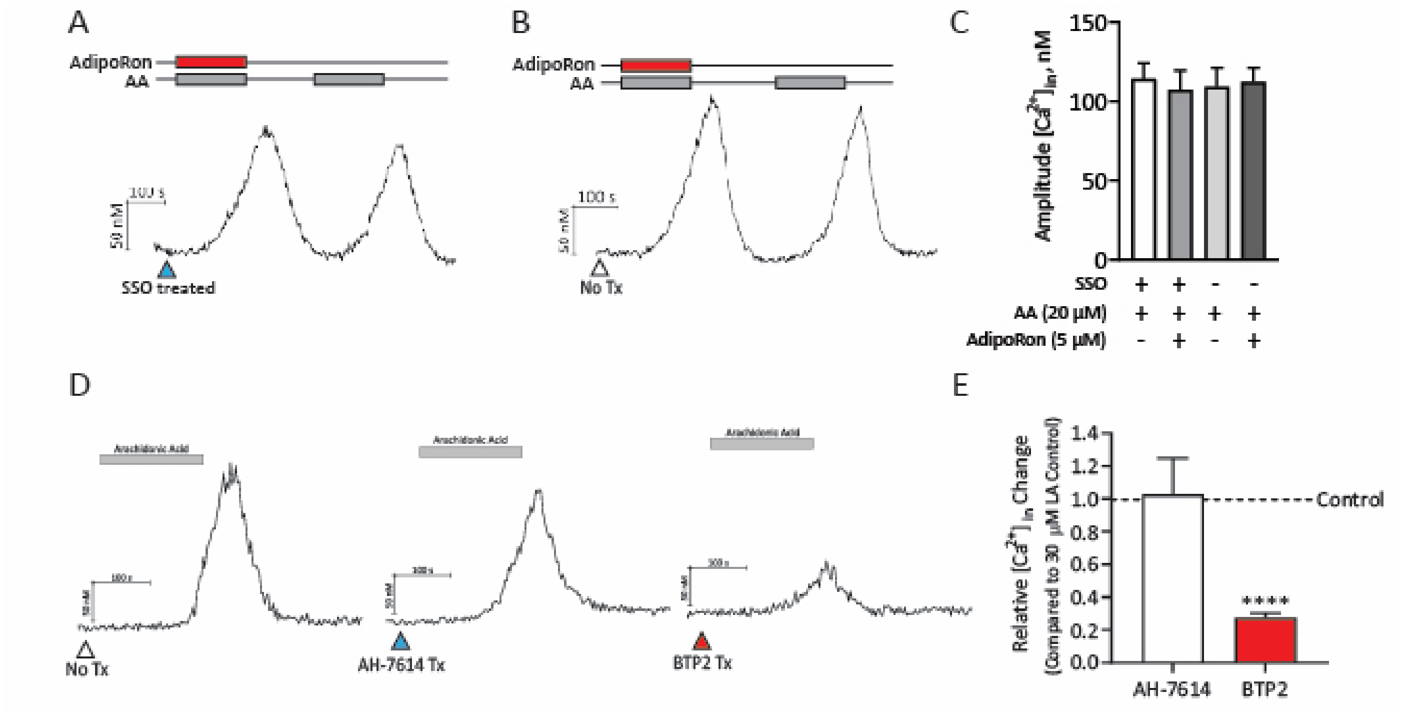

Figure S3. AA-induced cellular responses are not enhanced by AdipoRon in HUFF cells. Representative calcium trace showing responses to 20 μM AA with or without 5 μM AdipoRon in SSO pre-treated (***A***) or untreated (***B***) HuFF cells. ***C***, Effect of SSO, AdipoRon, and SSO + AdipoRon on calcium responses to 20 μM AA. Unlike the case for other PUFAs, AdipoRon and SSO had no effect on AA-induced responses. ***D***, Representative calcium trace showing responses to 20 μM AA in no treatment, AH-7614 pre-treated, and BTP2 pre-treated HuFF cells. ***E***, Effect of AH-7614 and BTP2 on calcium responses to 20 μM AA. The GPR120 antagonist AH-7614 had no effect on AA-induced responses, whereas BTP2, which inhibits store-operated calcium entry, inhibited AA-induced responses. Data are presented as mean ± SEM. Unpaired student's t-tests were used to determine statistical significance (**** $p < 0.0001$).
